# Assessment of Myocardial Dysfunction by Three-Dimensional Echocardiography Combined With Myocardial Contrast Echocardiography in Type 2 Diabetes Mellitus

**DOI:** 10.3389/fcvm.2021.677990

**Published:** 2021-06-07

**Authors:** Wei Li, Xiao-zhou Lv, Jia Liu, Jia-hui Zeng, Min Ye, Cui-ling Li, Rui Fan, Hong Lin, Hui-ling Huang, Feng-juan Yao

**Affiliations:** ^1^Department of Medical Ultrasonics, The First Affiliated Hospital of Sun Yat-sen University, Guangzhou, China; ^2^Department of Traditional Chinese Medicine, The First Affiliated Hospital of Sun Yat-sen University, Guangzhou, China; ^3^Department of Cardiology, The First Affiliated Hospital of Sun Yat-sen University, Guangzhou, China

**Keywords:** diabetes mellitus, myocardial dysfunction, three-dimensional echocardiography, myocardial contrast echocardiography, microvascular complications

## Abstract

**Background:** We aimed to explore the value of combining real-time three-dimensional echocardiography (RT-3DE) and myocardial contrast echocardiography (MCE) in the left ventricle (LV) evaluating myocardial dysfunction in type 2 diabetes mellitus (T2DM) patients.

**Patients and Methods:** A total of 58 T2DM patients and 32 healthy individuals were selected for this study. T2DM patients were further divided into T2DM without microvascular complications (*n* = 29) and T2DM with microvascular complications (*n* = 29) subgroups. All participants underwent RT-3DE and MCE. The standard deviation (SD) and the maximum time difference (Dif) of the time to the minimum systolic volume (Tmsv) of the left ventricle were measured by RT-3DE. MCE was performed to obtain the perfusion measurement of each segment of the ventricular wall, including acoustic intensity (A), flow velocity (β), and A·β.

**Results:** There were significant differences in all Tmsv indices except for Tmsv6-Dif among the three groups (all *P* < 0.05). After heart rate correction, all Tmsv indices of the T2DM with microvascular complications group were prolonged compared with the control group (all *P* < 0.05). The parameters of A, β, and A·β for overall segments showed a gradually decreasing trend in three groups, while the differences between the three groups were statistically significant (all *P* < 0.01). For segmental evaluation of MCE, the value of A, β, and A·β in all segments showed a decreasing trend and significantly differed among the three groups (all *P* < 0.05).

**Conclusions:** The RT-3DE and MCE can detect subclinical myocardial dysfunction and impaired myocardial microvascular perfusion. Left ventricular dyssynchrony occurred in T2DM patients with or without microvascular complications and was related to left ventricular dysfunction. Myocardial perfusion was reduced in T2DM patients, presenting as diffuse damage, which was aggravated by microvascular complications in other organs.

## Introduction

Type 2 diabetes mellitus (T2DM), characterized by hyperglycemia, is an independent risk factor for cardiovascular disease (1, 2). Cardiovascular consequences of diabetes are atherosclerotic epicardial coronary artery disease (CAD) and cardiomyocyte and myocardium changes. Patients with T2DM have a 2–4-fold increased risk of cardiovascular events (3). One major cause of increased mortality in T2DM is diabetic cardiomyopathy (DCM), which is defined as myocardial dysfunction that is independent of CAD and hypertension and can lead to heart failure (4, 5). Therefore, early detection of myocardial dysfunction in patients with T2DM is essential for therapeutic interventions that may prevent or reverse DCM, as the severity of cardiac disease determines prognosis.

Before clinically manifesting heart disease, patients with T2DM develop subtle changes in cardiac function such as left ventricular (LV) myocardial mechanical movement dysfunction including diastolic and systolic dysfunction (6, 7). Echocardiography is a currently available method for simultaneously evaluating myocardial morphology, motion, and perfusion. Two-dimensional echocardiography (2DE) and tissue Doppler-based techniques have shown variable results in the assessment of global LV systolic and diastolic function of T2DM (8, 9). Several parameters derived from Doppler tissue imaging (DTI) have been reported to be promising predictors of LV reverse remodeling (10). However, these indexes do not reflect the motion pattern of all LV segments, and because of the limitation of angle dependence of DTI, information regarding the apical segments cannot be evaluated. Real-time three-dimensional echocardiography (RT-3DE) is an appealing novel imaging modality that allows simultaneous assessment of the entire LV from a single full-volume data set acquired from the apex (11). In addition, this semi-automated software uses a 17-segment model of the heart to provide regional volume vs. time curves, which can be used to assess LV dyssynchrony (11, 12).

Except for LV dyssynchrony, impaired coronary microvasculature might lead to myocardial dysfunction in T2DM (13, 14). Myocardial contrast echocardiography (MCE) is a non-invasive method for assessing myocardial perfusion by observing destruction and replenishment of microbubbles into the myocardium (15). A previous study has shown that T2DM patients have impaired MCE-derived quantitative myocardial perfusion parameters compared to non-diabetic patients. Besides, various microvascular disease states are associated with risk of vascular disease, including cardiac autonomic neuropathy, retinopathy, nephropathy, and peripheral neuropathy (16–19).

Therefore, the current study aimed to quantitatively evaluate LV myocardial dyssynchrony using RT-3DE and myocardial perfusion by MCE in T2DM patients with microvascular complications and investigate the association between LV subclinical myocardial dysfunction and coronary microvascular perfusion.

## Patients and Methods

### Study Population

The study was approved by the Institutional Review Board of our hospital with written informed consent obtained from all study participants. From October 2018 to May 2019, we prospectively recruited 58 patients with T2DM from in-patients attending the Department of Endocrinology at our institution. Diabetes was defined by a fasting plasma glucose of at least 7.0 mmol/L (126 mg/dl), random plasma glucose of at least 11.1 mmol/L (200 mg/dl), or the use of glucose-lowering medications, based on recommendations from the American Diabetes Association (20). Briefly, classification of T2DM was performed according to the following criteria: specific diagnostic code for T2DM (Read code C10F; ICD-10 code E11) with no contradictory code and patients with a diagnosis of diabetes at the age of 35 years or older with no insulin prescription within 1 year of diagnosis. All patients had no evidence of coronary heart disease or heart failure on the basis of history, 12-lead electrocardiograph (ECG), a normal ejection fraction on echocardiography, and metabolic exercise testing. The exclusion criteria were as follows: clinical evidence of CAD and previous myocardial infarction, primary cardiomyopathy, congenital heart disease, valvular stenosis or more than mild regurgitation, prosthetic valves or pacemakers, uncontrolled hypertension (systolic blood pressure >160 mmHg), and significant renal impairment (estimated glomerular filtration rate <30 ml/min). Importantly, all patients were initially screened for obstructive epicardial CAD (>50% of luminal stenosis) with coronary computed tomographic angiography (CCTA) or coronary angiography (CAG). Subjects were divided into two groups based on the presence or absence of at least one of the microvascular complications of diabetes: retinopathy, nephropathy, and peripheral neuropathy (21, 22). The diagnosis of microvascular complications should meet one of the following criteria: (1) nephropathy was defined as microalbuminuria (a moderate increase in albuminuria: 3–30 mg/mmol, 30–300 mg/g, 30–300 mg per 24 h, or reagent strip urinalysis) or an estimated glomerular filtration rate (eGFR) <60 ml/min per 1.73 m^2^; (2) diabetic retinopathy is defined as a spectrum of retinal microvascular lesions seen on retinal examination (23); and (3) peripheral neuropathy diagnosed by clinical symptoms, physical examination, and neurophysiological examinations, excluding primary neurological diseases. A control group of 32 age-, gender-, and body mass index-matched healthy participants were recruited. All healthy volunteers had no history of cardiac disease or diabetes mellitus and underwent laboratory measurements before enrollment. The exclusion criteria were impaired fasting glucose (fasting glucose >6.1 mmol/L) or hypertension (blood pressure >140/90 mmHg).

### Baseline Characteristics and Laboratory Analysis

The height and weight of all participants were recorded, and body mass index (BMI) was calculated as weight (kg) divided by the square of height (m). The duration of diabetes was recorded as reported by the patient. Blood pressure was recorded as an average of three measurements in the right arm in a sitting position that were obtained after a 10-min resting period. Before echocardiography examination, blood samples were obtained from patients and controls for standard laboratory analysis, including plasma glucose, glycated hemoglobin, triglyceride, high-density lipoprotein cholesterol (HDL) cholesterol, and low-density lipoprotein cholesterol (LDL) cholesterol, according to standard procedures of the central clinical laboratory in our hospital.

### Standard 2D, Flow, and Tissue Doppler Echo Examination

Echocardiography was performed using a Philips EPIQ 7C ultrasound scanner (Philips Medical Systems, Best, Netherlands) equipped with a X5-1 phased array probe (operated frequency range, 1.6–3.2 MHz). Scanning was performed in the left lateral decubitus position, with ECG simultaneously recorded. Aortic sinus, ascending aorta, left atrial end-systolic anteroposterior diameter (LA), left ventricular end-diastolic anteroposterior diameter (LV), right ventricular end-diastolic anteroposterior diameter (RV), left ventricular ejection fraction (LVEF), and stroke volume (SV) were obtained.

### Real-Time 3D Echocardiography

The real-time 3D imaging was performed to obtain a pyramidal volume data set from the apical transducer position. Gain and compression controls and time-gain compensation settings were optimized to ensure image quality. The entire LV cavity was included within the pyramidal volume scan. Real-time 3D data sets were acquired using a wide-angle acquisition (93° × 80°) mode, in which four wedge-shaped subvolumes (93 × 20° each) were obtained from five consecutive cardiac cycles. Data were acquired from the apical four-chamber position during held end expiration. Acquisition was triggered to the R wave on the ECG of every cardiac cycle, resulting in a total acquisition time of five heartbeats.

### Myocardial Contrast Echocardiography

The ultrasound contrast agent was applied by continuous infusion of SonoVue (Bracco, Milan, Italy) intravenously. SonoVue was infused initially at a rate of 1 ml/min and adjusted thereafter to minimize attenuation. Gain was optimized at the beginning of the study and held constant throughout. Focal depth and length were adjusted to cover the entire LV chamber. The focus was set at the level of the mitral annulus. A low mechanical index (MI) was used at 0.18 to guarantee that microbubbles were not destroyed. After the myocardium was filled with the contrast agent, a burst of high MI at 1.3 was triggered for five cardiac cycles to destroy contrast microbubbles within the myocardium. Then, the system was automatically converted to the low-energy real-time radiography state. The refilling of contrast microbubbles within the myocardium was recorded from the apical four-, two-, and three-chamber view. Images collected from 15 consecutive cardiac cycles after providing high-energy pulse were turned into the cineloop, which was stored in disks for offline analysis.

### Image Analysis

All images were subsequently analyzed offline using QLAB analysis software (version 2.0, Philips Medical Systems, Cleveland, OH, USA) by two experienced physicians who were blinded to the clinical data. For 3D imaging, end-diastolic apical two- and four-chamber view cut planes were semiautomatically obtained from the pyramidal data set. Then, five anatomic landmarks were manually initialized, including two points to identify the mitral valve annulus and one point to identify the apex. After manual identification of these points, the program automatically identified the 3D endocardial surface using a deformable shell model. Adjustments to the automatic surface detection were performed when necessary. Thereafter, the end-systolic frame was selected by identifying the frame with the smallest LV cavity cross-sectional area in both apical views. After initialization, surface detection was then repeated on this frame to obtain end-systolic volumes. Finally, the computer algorithm automatically defined and traced the endocardial border in all frames of the cardiac cycle. “Casts” of the LV endocardium were then automatically obtained from which global LV volumes vs. time curves were derived. The LV was divided into 17 segments from apex to base, and curves depicting regional volumes over the cardiac cycle were obtained for each segment. From these regional volume curves (excluding the apical cap, segment 17), the regional ejection time, defined as the time interval between the R wave and minimal end-systolic volume (Tmsv), was automatically calculated. To assess systolic synchrony, the SD of the regional volume time curves was obtained using 16 segments (Tmsv16-SD); 12 segments, 6 basal and 6 middle segments (Tmsv12-SD); and 6 basal segments (Tmsv6-SD) in each patient. In addition to the Tmsv indices, the maximal difference of Tmsv was also calculated, generating the following additional indices of dyssynchrony: the maximal difference or range of Tmsv among 16 segments (Tmsv16-Dif); among the 6 basal and 6 middle segments (Tmsv12-Dif); and among the 6 basal segments (Tmsv6-Dif). To eliminate the influence of heart rate difference between different subjects, the above measurements were corrected for heart rate, which are Tmsv16-SD%, Tmsv12-SD%, Tmsv6-SD%, Tmsv16-Dif%, Tmsv12-Dif%, and Tmsv6-Dif%.

For MCE, LV 16-segment model recommended by the American Society of Echocardiography was used to analyze the collected images. The regions of interest located in the first post-flash end-systolic frame were automatically copied onto subsequent selected frames and manually realigned frame by frame to maintain a central point within the ventricular wall in the entire replenishment sequence. With region of interest (ROI, 5 × 5 mm) placed at the center of each segment, the refilling process of contrast microbubbles within the myocardium during 15 consecutive cardiac cycles after providing high-energy pulse was analyzed. Pixel intensity of each frame image was calculated frame by frame by a computer. The mean signal intensity (SI) was measured automatically at the ROIs. The segmental SI was plotted against time (t), and the subsequent refilling curve was fitted to the following exponential function: y(t) = A(1 – exp – βt) + C, where y is the acoustic intensity at a time of t, A is the plateau where acoustic intensity represents myocardial blood volume (MBV), and β represents the mean blood flow velocity in the intramyocardial vascular network. Myocardial perfusion (MBF) was calculated as the product of myocardial blood volume (A) and microvascular flow velocity (β) and analyzed for each coronary region.

### Statistical Analysis

Statistical analyses were performed with MedCalc (version 15.2.2, MedCalc Software, Mariakerke, Belgium). All continuous data were evaluated for normality using the Kolmogorov–Smirnov test and are presented as mean ± standard deviation or median (interquartile range). Homogeneity of variance was evaluated using the Levene's test. One-way analysis of variance (one-way ANOVA) was used to compare continuous variables as clinical characteristics, routine echocardiographic parameters, 3DE parameters, and perfusion parameters among controls, and T2DM without microvascular complication, and T2DM with microvascular complication, while least significant difference test was used to analyze the difference within the groups when the *P*-value of one-way ANOVA was <0.05. To compare the difference among the groups, analysis with Bonferroni correction was applied. The Kruskal–Wallis test was used to analyze parameters that did not conform to normality or show homogeneity of variance. Binary variables were analyzed using the chi-square test. Differences were considered statistically significant for *P* < 0.05.

## Results

### Patients and Baseline Characteristics

The demographic, clinical, and biochemical data of patients with T2DM and controls matched for age, gender, and BMI are shown in Table 1.

**Table 1:** Baseline characteristics of the study cohort.

**Characteristics**	**Control group (*n =* 32)**	**T2DM without microvascular complications group (*n =* 29)**	**T2DM with microvascular complications group (*n =* 29)**	***P*-value**
Age (years)	52.5 ± 12.9	58.0 ± 13.8	59.5 ± 9.0	0.087
Gender (male/female)	16/16	12/17	13/16	0.172
Diabetes duration (years)	–	8.8 ± 8.2	12.9 ± 7.4	0.008
Rest HR (bpm)	73.4 ± 9.8	84.8 ± 16.0	80.7 ± 10.5	0.002
SBP (mmHg)	120.8 ± 12.8	126.9 ± 15.8	130.6 ± 20.3	0.079
DBP (mmHg)	73.0 ± 10.2	77.8 ± 11.5	75.7 ± 11.2	0.249
BMI (kg/m^2^)	23.8 ± 3.2	23.8 ± 2.8	24.1 ± 2.9	0.872
Glycated hemoglobin (%)	5.6 ± 0.3	8.8 ± 2.3	9.0 ± 1.6	0.006
Plasma glucose (mmol/L)	4.8 ± 0.4	10.3 ± 4.5	11.9 ± 5.8	<0.001
Triglyceride (mmol/L)	1.6 ± 1.1	1.6 ± 1.3	1.8 ± 1.1	0.915
HDL (mmol/L)	1.3 ± 0.4	1.1 ± 0.3	1.1 ± 0.4	0.096
LDL (mmol/L)	3.3 ± 0.7	2.9 ± 0.9	2.9 ± 0.9	0.357
Aortic sinus (mm)	32.2 ± 3.3	33.0 ± 2.5	33.3 ± 3.5	0.418
Ascending aorta (mm)	30.8 ± 4.9	32.1 ± 3.4	32.4 ± 3.3	0.279
LA (mm)	34.3 ± 4.5	33.5 ± 2.8	35.7 ± 3.9	0.087
RV (mm)	21.1 ± 1.8	21.1 ± 1.8	21.0 ± 2.0	0.976
IVSd (mm)	9.7 ± 1.7	9.89 ± 1.17	10.3 ± 1.3	0.267
LVDd (mm)	48.0 ± 3.6	46.0 ± 4.8	45.2 ± 5.1	0.063
LVPWd (mm)	8.4 ± 1.3	8.79 ± 1.03	9.4 ± 0.8	0.003
LVEF (%)	70.7 ± 6.1	71.0 ± 5.4	70.4 ± 5.7	0.933
SV (ml)	76.1 ± 13.5	70.7 ± 20.2	66.2 ± 18.2	0.113

*Data are mean ± standard deviation or numbers*.

*HR, heart rate; SBP, systolic blood pressure; DBP, diastolic blood pressure; BMI, body mass index; HDL, high-density lipoprotein cholesterol; LDL, low-density lipoprotein cholesterol; LA, left atrial end-systolic anteroposterior diameter; RV, right ventricular end-diastolic anteroposterior diameter; IVSd, interventricular septal thickness; LVDd, left ventricular end-diastolic anteroposterior diameter; LVPWd, left ventricular posterior wall thickness at end-diastole; LVEF, left ventricular ejection fraction; SV, stroke volume*.

Diabetes duration, rest heart rate, glycated hemoglobin, plasma glucose, and left ventricular posterior wall thickness at end diastole (LVPWd) were significantly different among the three groups (all *P* < 0.05). Other parameters were not significantly different among the three groups (*P* > 0.05).

### Mechanical Dyssynchrony

**Figure 1:**
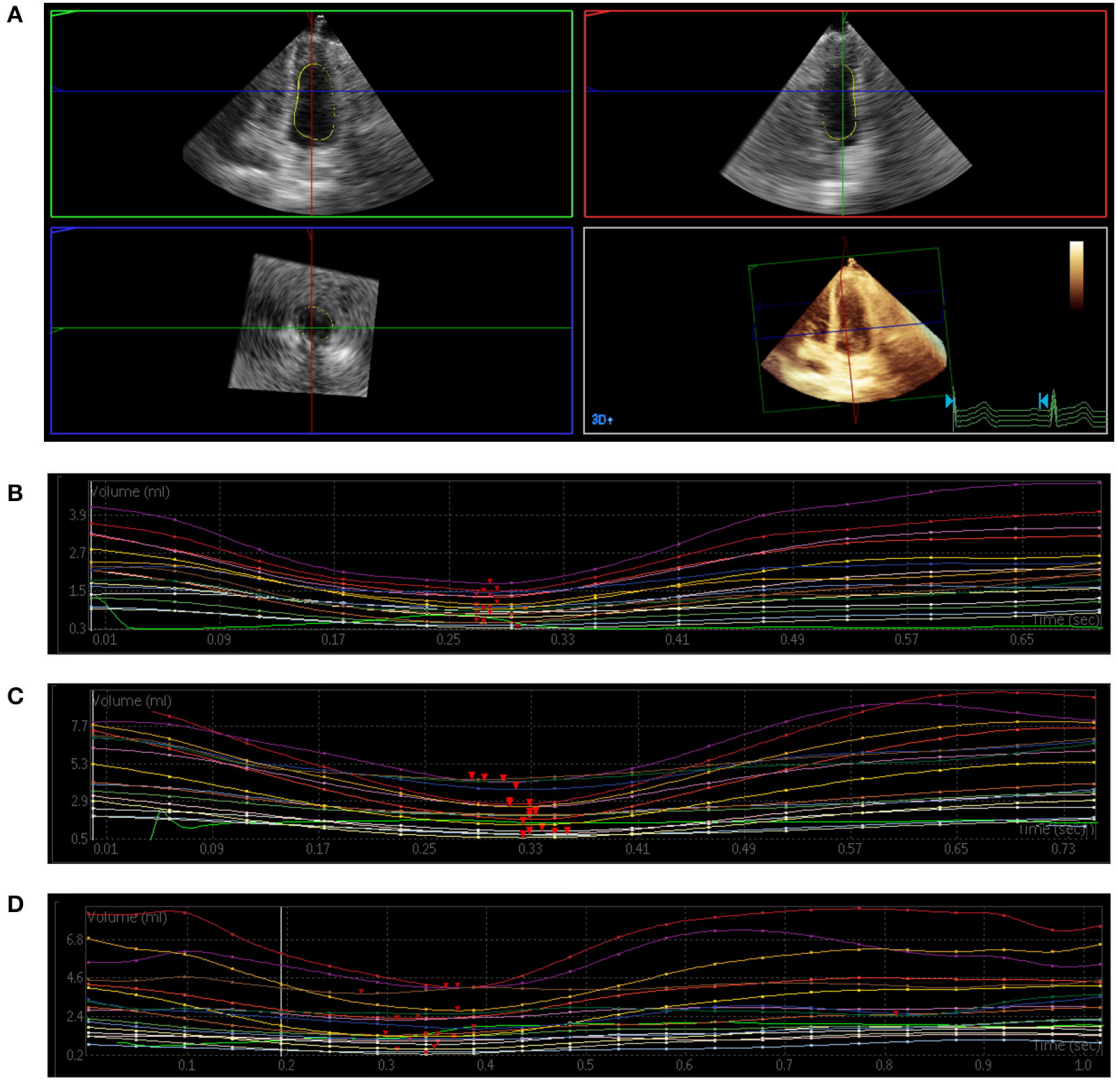
Three-dimensional echocardiography assessment of left ventricular (LV) mechanical dyssynchrony. **(A)** Full-volume acquisition. **(B)** Patient with normal intraventricular synchrony. There is a regular time–volume curves, and the time of reaching the trough is consistent. **(C)** Patient with slightly intraventricular dyssynchrony. The time–volume curves is regular but not consistent. **(D)** Patient with severe intraventricular dyssynchrony. There is a disarray of the time–volume curves signifying LV dyssynchrony.

Figure 1 shows the examples obtained from three patients with RT-3DE-derived LV dyssynchrony indices and regional volume curves obtained in 16 segments. There were significant differences in all Tmsv indices except for Tmsv6-Dif among the three groups (all *P* < 0.05). Tmsv16-Dif, Tmsv16-SD, Tmsv12-Dif, Tmsv12-SD, and Tmsv6-SD of T2DM with microvascular complications group were prolonged compared with the control group (all *P* < 0.05). The Tmsv12-Dif and Tmsv12-SD of the T2DM without microvascular complications group were prolonged compared with the control group (both *P* < 0.05). The Tmsv16-Dif, Tmsv16-SD, Tmsv12-Dif, and Tmsv12-SD of the T2DM with microvascular complications group were prolonged compared with the T2DM without microvascular complications group (all *P* < 0.05).

After heart rate correction, all Tmsv indices of the T2DM with microvascular complications group were prolonged compared with the control group (all *P* < 0.05). Tmsv12-Dif% and Tmsv12-SD% of the T2DM without microvascular complications group were prolonged compared with the control group (both *P* < 0.05). All Tmsv indices of the T2DM with microvascular complications group were prolonged compared with the T2DM without microvascular complications group (all *P* < 0.05) (Table 2).

**Table 2:** Comparison of parameters of 3DE in three groups.

**Parameters**	**Control group**	**T2DM without microvascular complications group**	**T2DM with microvascular complications group**	***P-*value**
Tmsv16-Dif	79.3 ± 32.1	89.3 ± 30.4	126.1 ± 83.5^*^^#^	0.011
Tmsv16-Dif%	9.5 ± 4.4	10.7 ± 3.4	15.8 ± 9.5^*^^#^	0.002
Tmsv16-SD	20.7 ± 8.5	23.9 ± 7.9	33.5 ± 23.1^*^^#^	0.013
Tmsv16-SD%	2.5 ± 1.2	3.0 ± 0.9	4.3 ± 2.7^*^^#^	0.003
Tmsv12-Dif	59.2 ± 29.4	73.6 ± 24.1^*^	111.7 ± 84.7^*^^#^	0.004
Tmsv12-Dif%	7.2 ± 4.1	8.8 ± 2.8^*^	14.1 ± 9.8^*^^#^	0.001
Tmsv12-SD	17.7 ± 9.2	22.4 ± 7.5^*^	33.5 ± 26.4^*^^#^	0.007
Tmsv12-SD%	2.2 ± 1.4	2.8 ± 0.9^*^	4.2 ± 3.0^*^^#^	0.002
Tmsv6-Dif	48.4 ± 28.3	53.1 ± 25.7	64.0 ± 65.5	0.06
Tmsv6-Dif%	5.8 ± 3.7	7.3 ± 3.1	10.6 ± 8.0^*^^#^	0.010
Tmsv6-SD	18.7 ± 11.8	23.3 ± 9.8	32.5 ± 24.9^*^	0.022
Tmsv6-SD%	2.2 ± 1.5	2.9 ± 1.2	4.1 ± 3.2^*^^#^	0.013

*Data are mean ± standard deviation*.

* *compared to control group, P < 0.05*.

# *compared to T2DM without microvascular complications group, P < 0.05*.

### MCE

**Figure 2:**
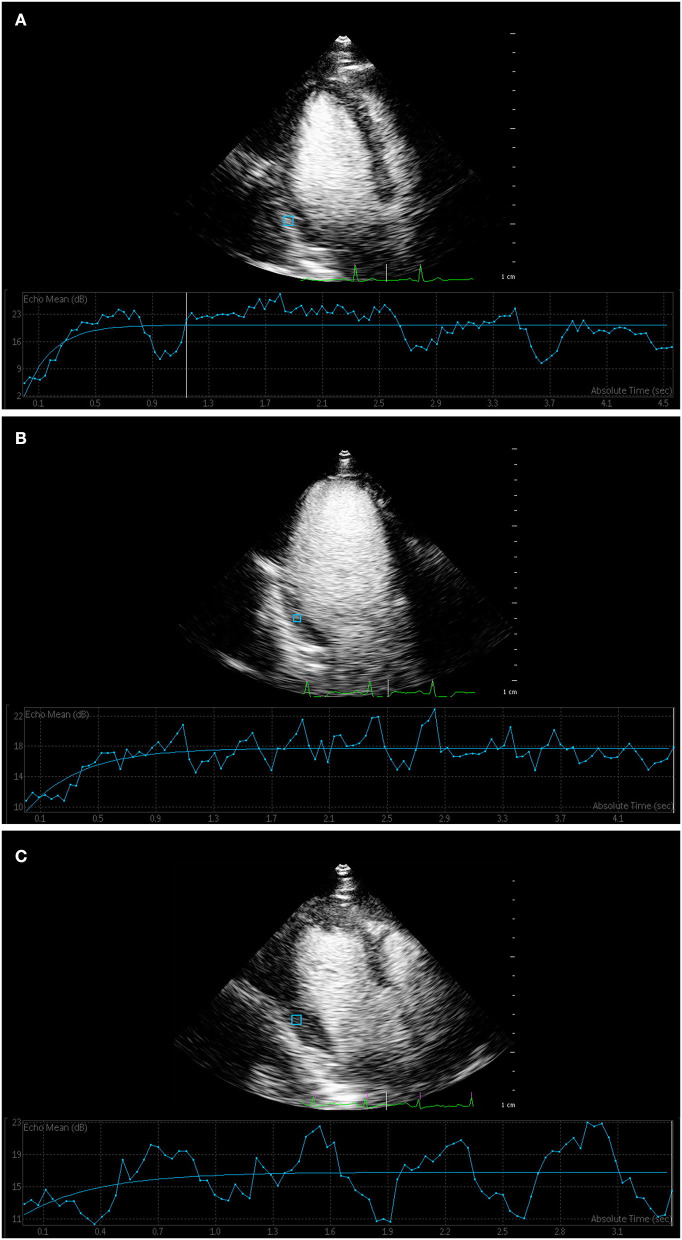
Myocardial contrast echocardiography of myocardial perfusion. **(A)** Time–intensity curve showed that the curve of control group rose rapidly to the plateau stage, and the peak intensity was high. **(B)** Time–intensity curve of T2DM without microvascular complications group and **(C)** T2DM with microvascular complications group increased slowly, and peak intensity was lower than that of control group.

The parameters of quantitative MCE among the three groups were compared, and the results are shown in Table 3. gThe parameters of A, β, and A·β for overall segments showed a gradually decreasing trend in three groups, while the differences between the three groups were statistically significant (*P* < 0.01) (Figure 2). All parameters of T2DM with microvascular complications group were significantly lower than the control and T2DM without microvascular complications groups (*P* < 0.05). Compared with the control group, the values of β and A·β in the T2DM without microvascular complications group decreased, both of which were statistically significant (*P* < 0.05).

**Table 3:** Comparison of MCE parameters in three groups.

**Parameters**	**Control group**	**T2DM without microvascular complications group**	**T2DM with microvascular complications group**	***P*-value**
**Peak Intensity (dB)**				
Overall segments	22.9 ± 10.8	18.4 ± 8.5	14.0 ± 7.4^*^^#^	0.001
Basal segments	19.6 ± 9.8	16.9 ± 10.1	12.3 ± 6.8^*^	0.010
Middle segments	24.3 ± 12.3	19.9 ± 9.2	15.3 ± 8.2^*^	0.004
Apical segments	24.9 ± 11.2	18.6 ± 8.0^*^	14.3 ± 8.4^*^	<0.001
**Time to peak (s)**				
Overall segments	5.8 ± 1.8	8.2 ± 2.1^*^	9.7 ± 2.4^*^^#^	<0.001
Basal segments	5.5 ± 2.0	8.0 ± 2.2^*^	9.7 ± 2.6^*^^#^	<0.001
Middle segments	6.0 ± 1.8	8.2 ± 2.0^*^	9.5 ± 2.4^*^^#^	<0.001
Apical segments	6.0 ± 1.8	8.4 ± 2.2^*^	10.0 ± 2.4^*^^#^	<0.001
**Wash in slope (dB/s)**				
Overall segments	10.3 ± 2.5	6.6 ± 3.2^*^	4.0 ± 1.6^*^^#^	<0.001
Basal segments	11.1 ± 3.6	6.6 ± 4.2^*^	3.8 ± 1.9^*^^#^	<0.001
Middle segments	10.6 ± 3.5	7.1 ± 2.7^*^	4.7 ± 2.0^*^^#^	<0.001
Apical segments	9.2 ± 2.8	6.2 ± 3.7^*^	3.5 ± 1.9^*^^#^	<0.001
**A × k**				
Overall segments	250.3 ± 145.2	142.0 ± 117.2^*^	63.6 ± 48.9^*^^#^	<0.001
Basal segments	220.6 ± 143.1	135.2 ± 142.1^*^	52.5 ± 47.2^*^^#^	<0.001
Middle segments	281.5 ± 187.9	152.8 ± 113.7^*^	79.0 ± 59.0^*^^#^	<0.001
Apical segments	248.8 ± 144.9	138.0 ± 132.6^*^	59.2 ± 55.2^*^^#^	<0.001

*Data are mean ± standard deviation*.

* *compared to control group, P < 0.05*.

# *compared to T2DM without microvascular complications group, P < 0.05*.

For segmental evaluation of MCE, the value of A, β, and A·β in all segments showed a decreasing trend and significantly differed among the three groups (*P* < 0.05). A, β, and A·β in all segments of the T2DM with microvascular complications group decreased compared with the control group (*P* < 0.05); β and A·β in all segments of the T2DM with microvascular complications group decreased compared with the T2DM without microvascular complications group (*P* < 0.05). Compared with the control group, the values of β and A·β in the basal segments, middle segments, and apical segments significantly decreased in the T2DM without microvascular complications group (*P* < 0.05).

## Discussion

Diabetic cardiovascular complications such as myocardial infarction and congestive heart failure are one of the main causes of death in T2DM patients (24, 25). Therefore, it is important to detect early LV dysfunction and treat it properly to reduce the long-term mortality of diabetic patients. LV dyssynchrony and impaired coronary microvasculature might lead to myocardial dysfunction in T2DM patients. RT-3DE is an appealing novel imaging modality that has been recently used in quantitative evaluation of global and regional LV mechanical movement function and the severity of cardiac dyssynchrony. MCE is a non-invasive method for quantitatively assessing myocardial perfusion and coronary microvasculature. Therefore, accurate assessment of myocardial dysfunction should contain the synchronization of myocardium mechanical movement and microvascular perfusion through combining with RT-3DE and MCE.

In this study, we found that LVEF and the size of the atrium and ventricle obtained by 2D echocardiography was comparable between the control and T2DM groups, and all the measured values were within a normal range, meaning that the global change of heart did not appear. After heart rate correction, all Tmsv indices obtained through RT-3DE of the T2DM with microvascular complications group were prolonged compared with the control and T2DM without microvascular complications groups (*P* < 0.05). The T2DM with microvascular complication group had significantly longer Tmsv indices. We could find that all synchronization indicators of T2DM with microvascular complications group, except for Tmsv6-Dif, were prolonged compared with the control group (*P* < 0.05), which indicates that when the conventional cardiac function measurement is within the normal range, the left ventricle of patients with T2DM exhibits asynchronized myocardial segmental contraction. Myocardial damage in patients with long-term T2DM has been confirmed by several studies, which agrees with the results of this study. The Tmsv12-Dif, Tmsv12-SD, Tmsv12-Dif%, and Tmsv12-SD% of the T2DM without microvascular complications group were also prolonged compared with the control group (*P* < 0.05), suggesting that incoordination of left ventricular movement in T2DM patients with complications is more obvious than in patients without other organ complications. One possible reason is that long-term hyperglycemia causes endothelial cells and intima fibrosis, as well as thickening of capillary basement membrane, which gradually progress to myocardial atrophy and fibrosis. These alterations lead to changes in hemorheology, leading to long-term myocardial ischemia and hypoxia and modifications in ventricular morphology and structure, which are finally manifested in the reduction in the left ventricular systolic function. In addition to the increase in blood glucose, the decline in renal excretion in patients with diabetic nephropathy also affects the structure and function of the left heart. Because glucose metabolism and lipid metabolites cannot be discharged in time, a large number of toxic products are introduced into the circulating blood, which is toxic to the heart, and the myocardium can be severely or permanently damaged, and thus, asynchrony of its myocardial movement becomes severe.

MCE is a technique for evaluating myocardial tissue microcirculation perfusion with a wide range of applications. It can be used for non-invasive and quantitative evaluation of myocardial microcirculation. The myocardial contrast agent does not infiltrate into the interstitial space during circulation. It can evaluate the microcirculation perfusion in real time, observe whether the myocardial blood perfusion is uniform, and detect the blood volume of the capillaries in the myocardium (26). The A, β, and A·β in the basal, middle, and apical segments of the T2DM with microvascular complications group decreased compared with the control group (*P* < 0.05); β and A·β in all segments of the T2DM with microvascular complications group decreased compared with the T2DM without microvascular complications group (*P* < 0.05). The overall myocardial contrast-enhanced ultrasound analysis showed that the A, β, and A·β of the T2DM group were lower than those of the control group, suggesting that the myocardial blood volume, blood flow velocity, and blood flow of T2DM patients were lower than those of the control group, which coincide with the results of studies using MRI to evaluate the microcirculation of T2DM. The parameters of A, β, and A·β in the T2DM with microvascular complications group were significantly lower than those in the T2DM without microvascular complications group (*P* < 0.05), suggesting that patients with T2DM have a certain degree of myocardial microcirculation disorder. With the microcirculation complications in other organs, the degree of reduction in various indicators of myocardial perfusion increased. In this study, the perfusion rate and local blood flow significantly decreased, which indicates that T2DM can cause systemic diffuse diseases, except for retinopathy and kidney damage, and the heart is also damaged. This may be attributable to impaired dilation of coronary microcirculation caused by hyperglycemia, which includes a decrease in the secretion of diastolic vasoactive substances such as nitric oxide and increase in the secretion of vasoconstrictor substances. Pathologically, the peri-capillary basement membrane is thickened, and vascular diameter is relatively narrow in T2DM patients (27). Vascular fibrosis contributes to arterial stiffening and reduces the compliance of vascular wall, increasing the risk of cardiovascular events (28). The number of capillaries per unit volume of the myocardium is relatively reduced. The combined effects of these factors promote an increase in local myocardial vascular resistance and decrease in blood volume. Segmental MCE analysis showed that there was no statistical difference among the three segments of the same index in the same group (*P* > 0.05), and the comparison of the basal, middle, and apical segments among the three groups showed a similar trend in the overall assessment. Perfusion velocity, peak intensity, and myocardial blood flow decreased in all segments, suggesting that impairment of myocardial microcirculation is diffuse instead of limited to specific segments in T2DM patients.

Our results demonstrated that (1) Tmsv and A, β, and A·β may be used to detect early LV dysfunction including mechanical dyssynchrony and decrease in myocardial perfusion in T2DM patients with microvascular complication; (2) LV myocardium is damaged, and the coordination and synchronization of myocardial movement are poor in T2DM patients; and (3) subclinical LV diastolic and systolic function were impaired in patients with T2DM. These phenomena may be explained by the following reasons. Various pathogeneses such as metabolic disorders, cardiomyocyte apoptosis, microvascular disease, oxidative stress, and mitochondrial structural disorders may be involved in myocardial hypertrophy and compliance reduction, LV remodeling, and ventricular wall rigidity, which may lead to damage to LV systolic and/or diastolic function.

Our study had several limitations. First, the number of samples in this study is small, and no further analysis of the factors affecting the various complications of T2DM has been carried out. Second, the control healthy patients did not undergo CCTA or CAG because of the radiation dose. However, all T2DM patients were initially screened with CCTA or CAG examination. Third, the MCE and 3DE highly depended on image quality and operator proficiency. For patients with poor acoustic window conditions, such as obesity, excessive lung gas, flat chest, etc., there are certain difficulties in acquiring satisfactory images; what is more, the 3DE technology may not be able to apply in patients with large heart chambers, as the entire LV chamber is hard to cover. Meanwhile, the patients with arrhythmia and breath holding difficulties are also limited. Finally, as this was a cross-sectional study, there are inherent design limitations, and our results remain to be verified by longitudinal studies in T2DM patients. We aim to accomplish this in our future research endeavors.

Diabetic cardiomyopathy is a dangerous complication of diabetes. Active prevention and treatment can effectively reduce the high mortality of diabetes patients due to cardiovascular complications. Some studies have found that early medical treatment such as angiotensin-converting enzyme inhibitors and β-receptor blockers and synchronized treatment or strict control of the blood glucose could effectively improve left ventricular diastolic and systolic function in T2DM patients. Early insulin treatment can improve the microcirculation structure and increase myocardial blood perfusion. The MCE of A and A·β could be used as important parameters for monitoring the therapeutic intervention. RT-3DE and MCE can increase the detection rate of systolic dysfunction and decrease perfusion before myocardial damage develops into cardiomyopathy. Early evaluation of cardiomyopathy has a certain significance for the treatment and prognosis of patients, and it can also be used as an effective, economical, and simple method for regular follow-up after treatment.

## Data Availability Statement

The raw data supporting the conclusions of this article will be made available by the authors, without undue reservation.

## Ethics Statement

The studies involving human participants were reviewed and approved by Institutional Review Board of the First Affiliated Hospital of Sun Yat-sen University. The patients/participants provided their written informed consent to participate in this study.

## Author Contributions

F-jY and H-lH contributed conception and design of the study. WL collected data of clinical trials and drafted the manuscript together with X-zL and J-hZ. MY, HL, and C-lL revised and edited the final version of the manuscript for important intellectual content. JL and RF gave the technical support. The methodology of this study was developed by WL and X-zL. WL and X-zL contributed equally for the acquisition of data, e.g., WL contributed in imaging examination, and X-zL contributed in patient collection. WL and X-zL performed data analysis together, including imaging analysis, computational, and statistical analysis, and edited the manuscript together. All authors read and approved the final manuscript.

## Conflict of Interest

The authors declare that the research was conducted in the absence of any commercial or financial relationships that could be construed as a potential conflict of interest.

## Abbreviations

RT-3DE: real-time three-dimensional echocardiography
MCE: myocardial contrast echocardiography
LV: left ventricular
2DE: two-dimensional echocardiography
T2DM: type 2 diabetes mellitus
SD: standard deviation
Dif: maximum time difference
Tmsv: minimum systolic volume
A: acoustic intensity
β: flow velocity
CAD: coronary artery disease
DCM: diabetic cardiomyopathy
DTI: Doppler tissue imaging
ECG: electrocardiograph
CCTA: coronary computed tomographic angiography
CAG: coronary angiography
eGFR: estimated glomerular filtration rate
BMI: body mass index
HDL: high-density lipoprotein
LDL: low-density lipoprotein
LA: left atrial end-systolic anteroposterior diameter
LV: left ventricular end-diastolic anteroposterior diameter
RV: right ventricular end-diastolic anteroposterior diameter
LVEF: left ventricular ejection fraction
SV: stroke volume
MI: mechanical index
ROI: region of interest
SI: signal intensity
MBV: myocardial blood volume
MBF: myocardial perfusion.
